# Supplementary material for: A DSG1 Frameshift Variant in a Rottweiler Dog with Footpad Hyperkeratosis
Source: Genes (Basel). 2020 Apr 24;11(4):469. doi: 10.3390/genes11040469 (PMC7230267; doi:10.3390/genes11040469)
Supplement: Supplementary file 1 [file genes-11-00469-s001.zip › Figure_S1.pdf]

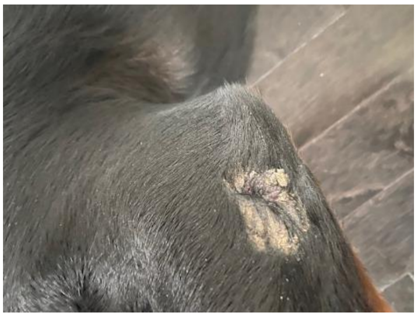

Figure S1. Hyperkeratotic plaque and crusting at the elbow of the affected dog, which was associated with bacterial infection of the skin in the affected dog.
